# PsrA controls the synthesis of the *Pseudomonas aeruginosa* quinolone signal via repression of the FadE homolog, PA0506

**DOI:** 10.1371/journal.pone.0189331

**Published:** 2017-12-08

**Authors:** Greg Wells, Samantha Palethorpe, Everett C. Pesci

**Affiliations:** Department of Microbiology and Immunology, The Brody School of Medicine at East Carolina University, Greenville, North Carolina, United States of America; Universite Paris-Sud, FRANCE

## Abstract

*Pseudomonas aeruginosa* is a ubiquitous, Gram-negative opportunistic pathogen that can cause disease in various sites within the human body. This bacterium is a major source of nosocomial infections that are often difficult to treat due to high intrinsic antibiotic resistance and coordinated virulence factor production. *P*. *aeruginosa* utilizes three cell-to-cell signaling systems to regulate numerous genes in response to cell density. One of these systems utilizes the small molecule 2-heptyl-3-hydroxy-4-quinolone (*Pseudomonas* quinolone signal [PQS]) as a signal that acts as a co-inducer for the transcriptional regulator PqsR. Quinolone signaling is required for virulence in multiple infection models, and PQS is produced during human infections, making this system an attractive target for potential drug development. In this study we have examined the role of a TetR-type transcriptional regulator, PsrA, in the regulation of PQS production by *P*. *aeruginosa*. Previous studies showed that PsrA regulates genes of the fatty acid β-oxidation pathway, including *PA0506*, which encodes a FadE homolog. In this report, we show that deletion of *psrA* resulted in a large decrease in PQS production and that co-deletion of *PA0506* allowed PQS production to be restored to a wild type level. We also found that PQS production could be restored to the *psrA* mutant by the addition of oleic or octanoic acid. Taken together, our data suggest that *psrA* positively affects PQS production by repressing the transcription of *PA0506*, which leads to a decrease in the conversion of acyl-CoA compounds to enoyl-CoA compounds, thereby allowing some octanoyl-CoA to escape the ß-oxidation pathway and serve as a PQS precursor.

## Introduction

*Pseudomonas aeruginosa* is a Gram-negative, ubiquitous bacterium and an important opportunistic human pathogen that causes chronic infections in both immunocompromised and cystic fibrosis (CF) patients [1, 2]. These infections have high morbidity and mortality rates due to intrinsic antibiotic resistance and the well-coordinated expression of a wide array of virulence factors [2, 3]. *P*. *aeruginosa* is very adaptable to diverse environments and is capable of surviving on many different carbon sources while generating a multitude of secondary metabolites [4], which in some cases affect virulence [5, 6]. This opportunistic pathogen also utilizes cell-to-cell signaling systems to regulate numerous genes, many of which are important for virulence [7, 8]. The LasRI and RhlRI quorum sensing systems respond to the acyl-homoserine lactone signals *N*-(3-oxododecanoyl)-L-homoserine lactone and *N*-butyryl-L-homoserine lactone, respectively [9, 10], while the quinolone signaling system functions through 2-heptyl-3-hydroxy-4-quinolone (*Pseudomonas* quinolone signal [PQS]) [11]. PQS serves as a coinducer for PqsR (also known as MvfR), which then transcriptionally activates the *pqsABCDE* operon to create a positive feedback loop that results in more PQS synthesis [12]. This feedback loop also leads to an increase in PqsE expression and this protein is required to mediate virulence through RhlR-C_4_-HSL via an unknown mechanism [13]. The first step in PQS synthesis is carried out by PqsA, which charges anthranilate with CoA to form athraniloyl-CoA [14]. Malonyl-CoA is then added via the action of PqsD, after which PqsB and PqsC catalyze the ligation of octanoyl-CoA to the newly formed 2-aminobenzoylacetate to create 2-heptyl-4-quinolone (HHQ) [15, 16]. The monooxygenase PqsH then catalyzes the formation of 2-heptyl-3-hydroxy-4quinolone (PQS) from HHQ [17]. An alternative product of the PQS synthesis pathway is 4-hydroxy-2-heptylquinoline *N*-oxide (HQNO) which is produced by the probable FAD-dependent monooxygenase, PqsL [14].

Previously, a study on factors that control the autolytic phenotype of *P*. *aeruginosa* demonstrated that deletion of *pqsL* caused an overproduction of PQS and colonies of this mutant grown on agar plates exhibited an autolytic phenotype [18]. Increased PQS levels in the *pqsL* mutant were attributed to a shift in the quinolone synthesis pathway because HQNO production stopped and more precursors for PQS synthesis were available. The study also identified ten other genes that affected PQS production [18]. One of these mutants harbored a disruption of gene *PA3006* (*psrA*), which is a transcriptional regulator belonging to the TetR family of regulators. PsrA directly activates the stationary phase sigma factor RpoS and also regulates genes involved in fatty acid metabolism in *P*. *aeruginosa*, including the *fadBA5* operon and *PA0506* [19–21]. Specifically, a prior microarray study suggested that *psrA* had a negative effect on the transcription of *PA0506*, a FadE homolog which is one of many predicted acyl-CoA dehydrogenases in strain PAO1 [21]. This enzyme is important for the conversion of acyl-CoA moieties (a PQS precursor) into enoyl-CoA during the first step of fatty acid β-oxidation. *P*. *aeruginosa* appears to have multiple sets of enzymes responsible for the breakdown of long chain fatty acids, including three *fadBA* operons which putatively encode a 3-hydroxyl-CoA dehydrogenase (FadB) and a 3-ketoacyl-CoA thiolase (FadA), which respectively catalyze the last two steps of fatty acid β-oxidation [22]. PsrA has been shown to bind to and repress the *fadBA5* and *PA0506* promoters at the C/GAAAC N_4_ GTTTG/C palindromic motif, and repression is relieved by long chain fatty acids [19, 21]. The fact that the loss of PsrA had a negative effect on PQS production suggested that the fatty acid β-oxidation pathway was important for PQS synthesis. In this study, we explore the link between PsrA and PQS production in *P*. *aeruginosa* and present data which suggest that negative regulation asserted by PsrA over the fatty acid β-oxidation pathway is required to allow acyl-CoA fatty acids to escape the β-oxidation pathway and be shuttled into quinolone production.

## Materials and methods

### Bacterial strains, plasmids, and culture conditions

All bacterial strains and plasmids are listed in Table 1. *E*. *coli* and *P*. *aeruginosa* strains were maintained in 10% glycerol and 10% Difco skim milk (Becton, Dickinson, and Co.), respectively, at -80°C. All strains were freshly plated to start each experiment and then grown at 37°C in Difco lysogeny broth (LB; Becton, Dickinson, and Co.). Growth was monitored spectrophotometrically based on the optical density at 660 nm (OD_660_) or 600 nm (OD_600_) for *P*. *aeruginosa* or *E*. *coli* strains, respectively. Strains were grown in the presence of either 200 μg/ml or 100 μg/ml carbenicillin (Research Products, Inc.) as needed to maintain plasmids for *P*. *aeruginosa* or *E*. *coli* strains, respectively. When needed, L-arabinose (0.5% final concentration) (Sigma-Aldrich) was added to cultures to induce genes controlled by the pBad promoter.

**Table 1:** Bacterial strains and plasmids used in this study.

Strain or Plasmid	Relevant genotype or phenotype	Reference or source
*E*. *coli* DH5α	λ^-^ φ80d*lacZ*ΔM15 Δ(*lacZYA-argF*) *U196 recA1 endA1 hsdR17* (r_K_^-^ m_K_^-^) *supE44 thi-1 gyrA relA1*	[23, 24]
*P*. *aeruginosa* strains		
PAO1	Wild type	[25]
PGW-Δ*psrA*	*psrA* deletion in PAO1	This Study
PGW-Δ*PA0506*	*PA0506* deletion PAO1	This Study
PGW-Δ*psrA*Δ*PA0506*	*PA0506* deletion in PGW-Δ*psrA*	This Study
PGW-Δ*PA0507*	*PA0507* deletion in PAO1	This Study
PGW-Δ*psrA*Δ*PA0507*	PA0507 deletion in PGW-Δ*psrA*	This Study
PGW-Δ*PA0508*	*PA0508* deletion in PAO1	This Study
PGW-Δ*psrA*Δ*PA0508*	*PA0508* deletion in PGW-Δ*psrA*	This Study
Plasmids		
pHERD20T	*E*. *coli/P*. *aeruginosa* shuttle expression vector	[26]
pEX18Ap	Suicide vector for *P*. *aeruginosa*	[27]
pEX1.8	Expression plasmid	[28]
pMW105	*rpoS* in pEX1.8	[29]
pPQSsynOE	P_BAD_-*pqsABCD* on pHERD20T	[30]
pGW-1	*psrA* deletion in pEX18Ap	This Study
pGW-2	P_BAD_-*psrA* on pHERD20T	This Study
pGW-14	PsrA-6HIS in pHERD20T	This Study
pGW-15	*PA0506* deletion in pEX18Ap	This Study
pGW-16	*PA0507* deletion in pEX18Ap	This Study
pGW-18	P_BAD_*-PA0507* on pHERD20T	This Study
pGW-19	*PA0508* deletion in pEX18Ap	This Study
pGW-20	P_BAD_*-PA0508 o*n pHERD20T	This Study
pGW-21	P_BAD_*-PA0506* on pHERD20T	This Study
pGW-22	P_BAD_*-fadAB5* on pHERD20T	This Study

### Generation of mutant strains and plasmids

All oligonucleotide primers are listed in S1 Table. Mutant *P*. *aeruginosa* strains were derived by a modified version of a previously published protocol [31]. The mutant alleles were generated by using a splicing-by-overlapping PCR extension of primers [32]. The alleles were constructed to harbor in-frame deletions in the DNA coding sequence corresponding to amino acids 18 to 226 for *psrA* (89% of protein sequence), 19 to 585 for PA0506 (94% of protein sequence), 36 to 553 for PA0507 (86% of protein sequence), and 38 to 535 for PA0508 (86% of protein sequence). Oligonucleotide primers utilized for this procedure were designed to generate at least 1000 bp of DNA upstream and downstream from the junction. Strain PAO1 chromosomal DNA was utilized as template for the PCR. The final PCR product was cloned into pEX18Ap using PstI-HF (*psrA*) and HindIII-HF (PA0506-08) (New England Biolabs) restriction enzymes, respectively.

Plasmids harboring Δ*psrA* (pGW-1), Δ*PA0506* (pGW-15), Δ*PA0507* (pGW-16), Δ*PA0508* (pGW-19) were sequenced to confirm in-frame deletions and transformed into strain PAO1 by electroporation [33, 34]. Mutants were selected as described by Hoang et al. [27]. Potential mutant colonies containing a *psrA* deletion in strain PAO1 (PGW-*ΔpsrA*) were screened by PCR utilizing appropriate flanking primers and confirmed by DNA sequencing of the PCR product. Plasmids pGW-15, pGW-16, and pGW-19 were transformed into strain PAO1 to generate single mutants, or strain PGW-Δ*psrA* to generate double mutants.

To generate expression plasmids for complementation experiments, genes *psrA* (702 bp), *PA0506* (1821 bp), *PA0507* (1796 bp), *PA0508* (1778 bp), and *fadAB5* (3354 bp) were amplified by PCR from strain PAO1 chromosomal DNA. The oligonucleotide primers with engineered restriction enzyme sites were utilized to harbor both the start codon and the stop codon for all genes. After digesting with restriction enzymes, the PCR fragment was ligated into the digested pHERD-20T which contains an *araC pBAD* (P_BAD_) promoter to control gene expression. These clonings resulted in plasmids pGW-2, pGW-21, pGW-18, pGW-20, and pGW-22, respectively.

### Measuring PQS production

Washed cells from an overnight culture were used to inoculate fresh 10 ml cultures to an OD_660_ of 0.05. After 24 hours of growth at 37°C with vigorous shaking, 300 μl of each culture was extracted with 900 μl of acidified ethyl acetate as previously described [35]. One-half of the organic phase was completely evaporated at 37°C, and 50 μl of 1:1 acidified ethyl acetate-acetonitrile was used to reconstitute the extract. Samples were analyzed by thin-layer chromatography (TLC), visualized by long-wave UV light, photographed, and quantified by densitometric analysis [35].

### Measuring pyocyanin production

Pyocyanin measurements were completed using a modified protocol from Essar et al. [36]. Washed cells from an overnight culture were used to inoculate 10 ml of LB media to an OD_660_ of 0.05, which were incubated at 37°C with vigorous shaking. After 24 hours of growth, cultures were centrifuged to remove bacterial cells and 500 μl of culture supernatant was extracted with 300 μl of chloroform. The organic phase was extracted with 100 μl of 0.2 N HCL, which produced a pink solution containing pyocyanin. The absorbance of this solution at 520 nm was measured using a NanoDrop ND-1000 spectrophotometer. Data are presented in absorbance as the mean ± standard deviation [σ^(n-1)^] of three independent experiments.

### Monitoring fatty acid effects of PQS

Octanoic acid or oleic acid (Sigma-Aldrich) was added to a final concentration of 10 mM to 10 ml of LB media and the pH was adjusted to 7.0 with sodium hydroxide. Flasks were inoculated with strain PAO1, PGW-Δ*psrA*, PGW-Δ*PA0506*, PGW-Δ*PA0507*, PGW-Δ*PA0508*, or PGW-Δ*psrA*, *0506* and incubated at 37°C with vigorous shaking overnight. Washed cells from these cultures were utilized to inoculate fresh 10 ml cultures containing the same fatty acid to an OD_660_ of 0.05 and incubated at 37°C with vigorous shaking. After 24 hours of growth, PQS was extracted and quantified as described above.

### Purification of PsrA

Overnight cultures of *E*. *coli* strain DH5α harboring the control plasmid (pHERD20T) or the expression vector (pGW-14) that contains a 6-His-tag-PsrA fusion protein were subcultured with a starting OD_600_ of 0.05. Cultures were incubated at 37°C with vigorous shaking for 2.5 hours to an OD_600_ of approximately 0.5, and L-arabinose was added to a final concentration of 0.5% to induce expression of PsrA. The cultures were then incubated for 3 hours and cells were harvested by centrifugation at 6,000 × g for 10 min at 4°C. Bacterial cell pellets were resuspended in 1 ml of STE buffer (pH 7.5; 10 mM Tris-HCl, 1 mM EDTA, 100 mM NaCl), and this suspension was passed through a French pressure cell at 16,000 lb/in^2^ to yield a whole-cell lysate. The 6-His-tag-PsrA protein was purified via Ni-NTA agarose (Qiagen) utilizing the native protein methods recommended by Qiagen. Protein concentration for DNA mobility shift assays was determined using a Bradford assay (Bio-Rad).

### DNA mobility shift assay

PCR was used to generate DNA fragments containing the *kynA* (228 bp), *psrA* (298 bp), *PA0506* (349-bp), *PA0507* (233-bp), or *PA0508* (342-bp) promoter region. DNA probes were labeled with ^32^P using [γ-^32^P] ATP (Perkin-Elmer) and T4 polynucleotide kinase (New England Biolabs). Binding reactions were carried out in buffer containing 20 mM HEPES (pH 7.6), 1mM EDTA, 10 mM ammonium sulfate, 150 mM potassium chloride, 1 mM DTT, and 5% glycerol. Each reaction mixture contained 0.3 μg of salmon sperm DNA, 3000 cpm of radiolabeled probe, and 0 to 50 ng of purified 6-His-tagged PsrA protein. Reaction mixtures were incubated at room temperature for 20 min and separated by electrophoresis on a native 6% polyacrylamide gel in 0.5X Tris-borate-EDTA buffer at 4°C. Gels were then exposed to X-ray film to visualize radiolabeled bands.

### RNA isolation

Overnight cultures of strains PAO1 and PGW-Δ*psrA* were washed in LB media and used to inoculate 10 ml of LB media to an OD_660_ of 0.05. Cultures were incubated at 37°C for 3 hours (or 6 hours for S2 Fig) or to an OD_660_ of approximately 1.5 prior to centrifugation at 4°C to harvest bacterial cells. Total cellular RNA was isolated from *P*. *aeruginosa* cells using the RNeasy Midiprep kit according to the manufacturer’s protocol (Qiagen). Contaminating DNA was removed by treatment of RNA samples with RQ1 DNase as per the manufacturer’s protocol (Promega). RNA was then extracted with 1:1 phenol-chloroform and then precipitated with ethanol. Purified RNA was resuspended in nuclease-free water. RNA concentration was determined with a NanoDrop ND-1000 spectrophotometer.

### cDNA synthesis for quantitative real-time PCR (qRT-PCR)

Total RNA was isolated from strains PAO1 and PGW-Δ*psrA*, and purified as described above. cDNA was synthesized in a 21 μl reaction volume from 5 μg of total RNA using a 1:1 mixture of GC-rich hexamers (Gene Link) and random hexamers (Invitrogen) for priming with 40 μM deoxynucleoside triphosphates (dNTPs) (USB). Reactions were then heated at 65°C for 5 min, followed by cooling to 4°C for 1 min. Following this step, 200 U of SuperScript III reverse transcriptase in First Strand buffer (Invitrogen) with 0.1 M dithiothreitol and RNase Out RNase inhibitor (Invitrogen) were added to each reaction mixture, yielding a final volume of 30 μl. Reaction mixtures were then heated to 25°C for 5 min, followed by 50°C for 1 hour and then 75°C for 15 min.

### qRT-PCR

cDNA and total RNA to be used as the template and negative control, respectively, were diluted 1:200 in nuclease-free water. Oligonucleotide primer pairs for qRT-PCR were generated by the Primer-BLAST program available at www.ncbi.nlm.nih.gov/tools/primer-blast/. Primers were designed to amplify a 200 bp fragment of *clpX* (control), a 132 bp fragment of *lasR*, a 150 bp fragment of *pqsA*, a 139 bp fragment of *PA0506*, a 124 bp fragment of *PA0507*, and a 117 bp fragment of *PA0508* as target genes. Quantitative RT-PCR was performed using FastStart SYBR green master mix (Roche Diagnostics) with Bio-Rad CFX96. The following cycle was utilized to amplify and quantify fragments: 95°C for 10 min and then 95°C for 15 s, 56°C for 15 s, and 72°C for 20 s, repeated 40 times. Melt curve data were collected to ensure amplification of one fragment by heating samples from 65°C to 95°C in 0.5°C increments. Data were generated from three separate RNA and cDNA preparations and at least two technical replicates for each primer set. Relative expression of each gene was determined by comparing target genes with the control gene (*clpX*) using the Pfaffl method [37].

### Reverse transcription polymerase chain reaction

DNase treated RNA (400 ng) from wild type strain PAO1 was used as a template for reverse transcriptase PCR (RT-PCR) performed utilizing the Promega AccessQuick RT-PCR system by following the manufacturer’s protocol. Primer pairs were designed to cover the intergenic regions of all genes of interest. All cDNA synthesis and PCR amplification steps were performed in an Eppendorf Mastercycler with the following parameters: cDNA synthesis at 45°C for 30 min; 95°C for 2 min; 30 cycles of 95°C for 45 s, 55°C for 45 s, and 72°C for 40 s. The final cycle was for 5 min at 72°C. Positive controls were performed using genomic DNA for strain PAO1 (100 ng), and negative controls were performed without adding reverse transcriptase. Reaction products were analyzed by agarose gel electrophoresis.

## Results

### PsrA positively controls PQS production in *P*. *aeruginosa*

**Fig 1:**
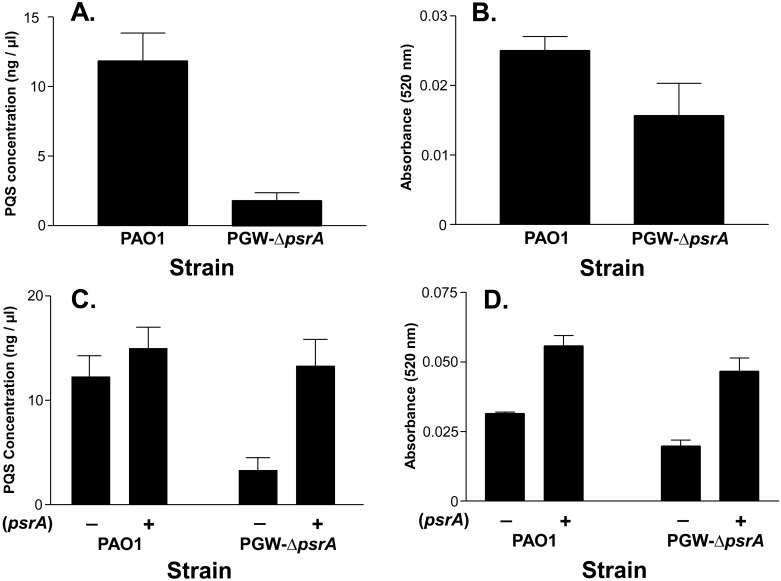
PsrA positively controls PQS production in *P*. *aeruginosa*. Strains PAO1 and PGW-Δ*psrA* were grown for 24 h in LB medium, then (A and C) PQS or (B and D) pyocyanin was extracted and quantified as described in Materials and Methods. The presence of *psrA* expression plasmid (pGW-2) or pHERD20T (control plasmid) is indicated by a plus or minus, respectively. For C and D, LB media was supplemented with 0.5% L-arabinose to induce *psrA*. All data are presented as the average ± SD of three independent experiments.

Our previous studies have shown that a *pqsL* mutant was autolytic and overexpressed PQS [18]. When this mutant was randomly mutagenized, a transposon insertion in *psrA* (PA3006) caused both the suppression of the autolytic phenotype and a decrease in PQS production [18]. To expand on this result, we constructed an isogenic *psrA* mutant (PGW-Δ*psrA*) and examined its phenotype. As expected, strain PGW-Δ*psrA* produced less PQS than the parent strain PAO1 (Fig 1A), and it also produced less of the PQS-controlled virulence factor pyocyanin (Fig 1B). PQS and pyocyanin production were both restored to at least the wild type level in strain PGW-Δ*psrA* containing a *psrA* expression plasmid (Fig 1C and 1D), indicating that mutation of *psrA* did not have polar effects on adjacent genes. Taken together, these data indicate that *psrA* positively controls PQS production.

### Expression of *pqsABCD* restores PQS activity in a *psrA* mutant

**Fig 2:**
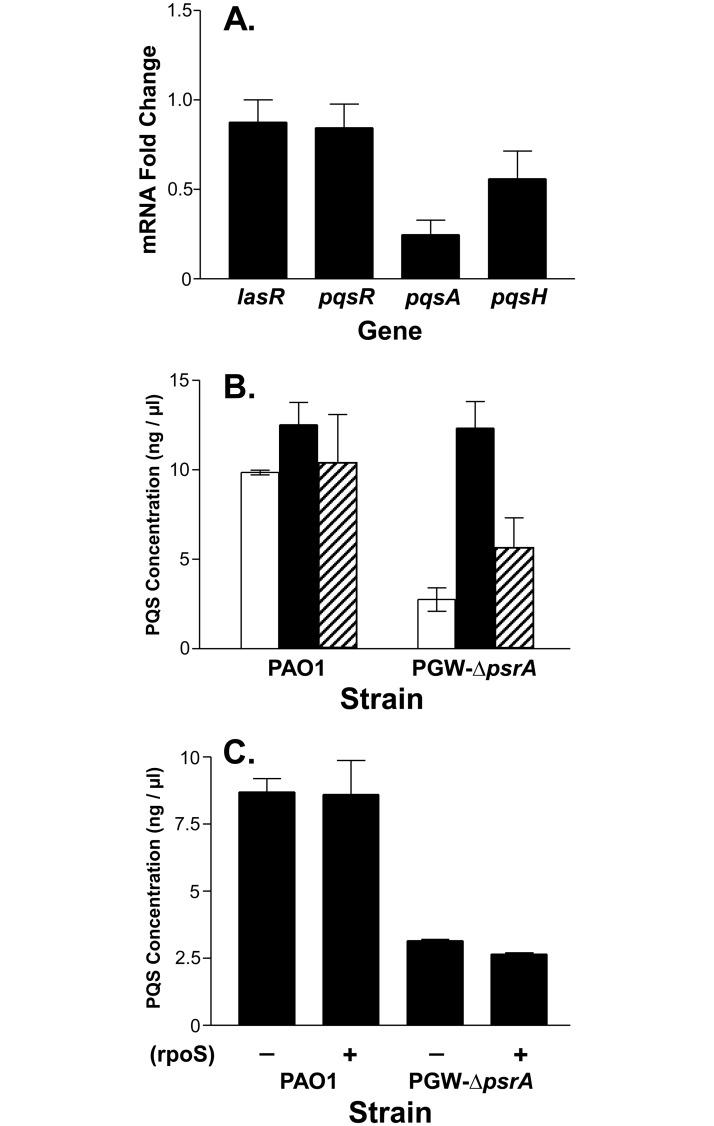
PsrA positively controls *pqsA*. (A) qRT-PCR was performed on RNA from strains PAO1 and PGW-Δ*psrA* to analyze the relative expression of *lasR*, *pqsH*, *pqsR* and *pqsA*. Data are presented as average fold change ± SD in the *psrA* mutant compared to the wild type strain (set to a value of 1). The gene *clpX* was used as a reference gene to normalize expression and each experiment was completely repeated three times. (B and C) PQS production by strains PAO1 or PGW-Δ*psrA* expressing (B) PQS synthetic genes or (C) RpoS. Cultures were grown for 24 h in LB medium supplemented with 0.5% L-arabinose to induce genes. PQS was then extracted and quantified as described in Materials and Methods. Data are presented as the average ± SD of three independent experiments. (B) Plasmids contained by strains are: open bars, control vector; solid bars, *pqsABCD* expression vector; and hatched bars, *pqsH* expression vector. (C) Presence of the *rpoS* expression vector is indicated by a plus (+) symbol.

Since PsrA has been shown to act as a transcriptional regulator that influences the expression of a wide range of genes, we decided to use qRT-PCR to assess the relative expression of genes required for PQS synthesis in a *psrA* mutant. LasR directly activates *pqsR* and *pqsH*, and PqsR directly activates *pqsA*, and the loss of any of these genes causes a drastic reduction in PQS production [12, 38, 39]. Our data showed that the expression of neither *lasR* nor *pqsR* was altered in the *psrA* mutant, indicating that the effect of *psrA* on PQS was not mediated through either of these two PQS-controlling regulators (Fig 2A). The expression of *pqsA* and *pqsH* decreased to 0.23 and 0.57, respectively, when compared to the parent strain PAO1 set to a value of 1 (Fig 2A). Both of these genes are directly involved in PQS synthesis and the *pqsABCDE* operon is positively controlled by PQS, so a decrease in the expression of *pqsA* would be expected if less PQS were present. This was confirmed by results which showed that expressing *pqsABCD* from an inducible promoter on a plasmid caused PQS production to return to a wild type level in the *psrA* mutant (Fig 2B). We also found that adding PQS to the *psrA* mutant containing a *pqsA-lacZ* reporter fusion caused *pqsA* expression to return to a wild type level (data not shown), indicating again that the observed effect of *psrA* was caused by lower PQS production in the *psrA* mutant. In addition, the overexpression of *pqsH* caused a small increase in PQS production in the *psrA* mutant to approximately half that of the wild type strain, suggesting that *pqsH* is not the mediator of the *psrA* effect on PQS production (Fig 2B).

We also noted from previous studies that PsrA was shown to have a positive effect on *rpoS* transcription, and that RpoS negatively regulates quorum sensing [19, 20, 29]. This led us to question whether the effect of PsrA on PQS production was mediated by RpoS. To test this, we examined PQS production in strain PGW-Δ*psrA* containing plasmid pMW105, which harbors an inducible *rpoS*. These data showed that RpoS had no effect on PQS production in the *psrA* mutant (Fig 2C), and eliminated this avenue of regulation as an explanation for the effect of *psrA* on PQS.

### PsrA regulates PQS production via the fatty acid β-oxidation cycle

**Fig 3:**
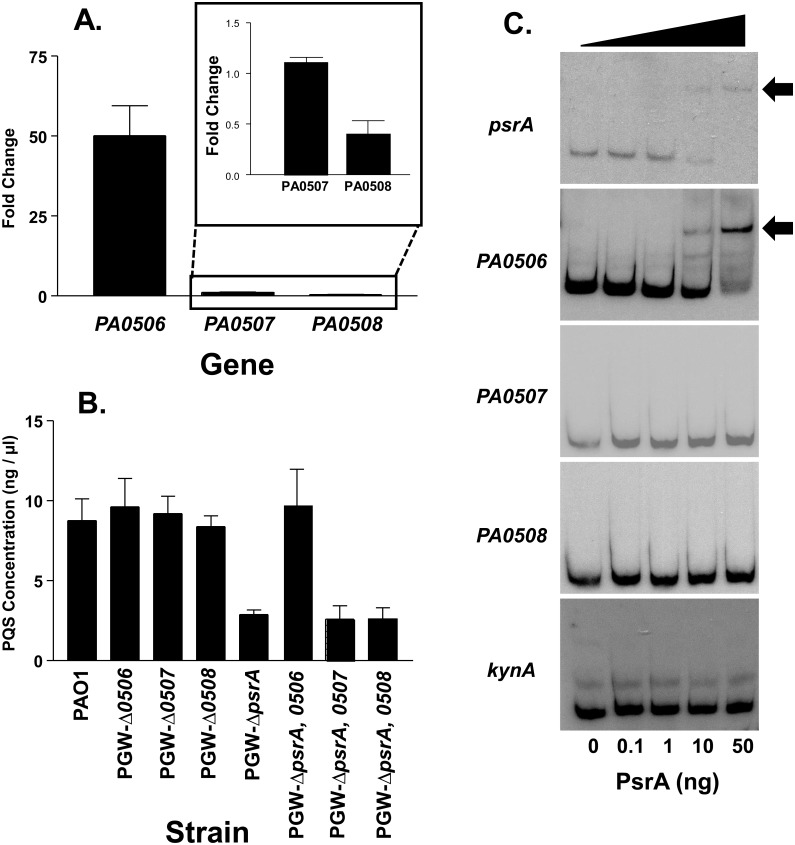
PsrA directly regulates *PA0506* to control PQS production. (A) qRT-PCR was performed on RNA from strains PAO1 and PGW-Δ*psrA*. Data are presented as average fold change ± SD of expression in the *psrA* mutant as compared to expression in the wild type strain (set to a value of 1). The gene *clpX* was used as a reference for normalization and experiments were repeated three separate times. Targeted genes are listed below the columns. (B) The indicated single and double mutants were assessed for PQS production after 24 h growth. (C) EMSA for promoter regions of *psrA* (positive control) [41], *PA0506*, *PA0507*, *PA0508*, and *kynA* (negative control) was performed using 0, 0.1, 1.0, 10, and 50 ng of PsrA-his tag protein. Binding is indicated by an arrow and data are representative of three independent experiments.

Several fatty acid β-oxidation enzymes have been identified in *P*. *aeruginosa* and there seems to be overlapping pathways and complex regulation designed to ensure that various fatty acids can be broken down when needed. For example, this organism has three putative *fadE* genes (*PA0506*, *PA0507*, and *PA0508*) which are adjacent to one another and encode acyl-CoA dehydrogenase homologs that would catalyze the first step of fatty acid β-oxidation. PsrA appears to be one of the major regulators of some fatty acid degradation enzymes as it has been shown to bind and repress both *PA0506* and the *fadBA5* operon, which is important for the breakdown of fatty acids with chain lengths of 12 or greater [19, 21, 40]. The repression of *fadBA5* by PsrA was relieved in the presence of fatty acids, with the repression decreasing as fatty acid chain length increased [21]. In addition, a prior microarray study suggested that *psrA* had a negative effect on *PA0506* and *PA0507* [21]. These reports, along with the fact that PQS production requires an octanoyl-CoA which can be derived from β-oxidation of long chain fatty acids, led us to test whether PQS concentrations were affected by one of the PsrA-regulated FadE homologs (*PA0506*, *PA0507*, and *PA0508*). We first attempted to confirm the prior microarray studies by using quantitative real time PCR to monitor the expression of *PA0506*, *PA0507*, and *PA0508* in a *psrA* mutant and its parent strain. This experiment showed that in the *psrA* mutant, *PA0506* expression was increased 50 fold as expected, *PA0507* expression was unchanged, and *PA0508* expression decreased to half that of the wild type strain (Fig 3A). The potential for differential regulation of these three genes was confirmed by reverse transcription-PCR, which showed that each gene is transcribed in a separate operon (S1 Fig). Since FadE catalyzes the first step in the fatty acid β-oxidation cycle, and PQS synthesis requires an octanoyl-CoA, we hypothesized that the overexpression of the FadE homolog PA0506 in the *psrA* mutant was probably causing a decrease in octanoyl-CoA available for PQS synthesis. To begin to test this, we created isogenic in-frame mutants of all three putative FadE homolog genes, *PA0506*, *PA0507*, and *PA0508*. While our results above suggested that a *PA0506* mutant might produce more PQS, we saw that all three mutants produced PQS similar to the wild type strain (Fig 3B). In retrospect, this was not really a surprise since the likely overlapping function of the three adjacent homologs probably negates any effects of a single mutant. To try to further establish the link between *psrA*, a FadE homolog, and PQS production, we also created and tested double mutants which had deletions of *psrA* and either *PA0506*, *PA0507*, or *PA0508* (Fig 3B). Analysis of PQS production by these three strains showed that the *psrA*, *PA0506* double mutant produced a wild type amount of PQS. This indicated that in order for *psrA* to control PQS production, *PA0506* had to be present. Removal of *PA0507* or *PA0508* in the *psrA* mutant had no effect on PQS production (Fig 3B). Furthermore, EMSA analysis confirmed that PsrA only binds to the *PA0506* promoter region (Fig 3C). Taken together, our data suggest that PQS production is controlled by PsrA via its negative regulation of *PA0506*, which prevents or slows down the fatty acid β-oxidation cycle of *P*. *aeruginosa* to allow the escape of the octanoyl-CoA needed for PQS synthesis.

### Long chain fatty acids induce PQS production

**Fig 4:**
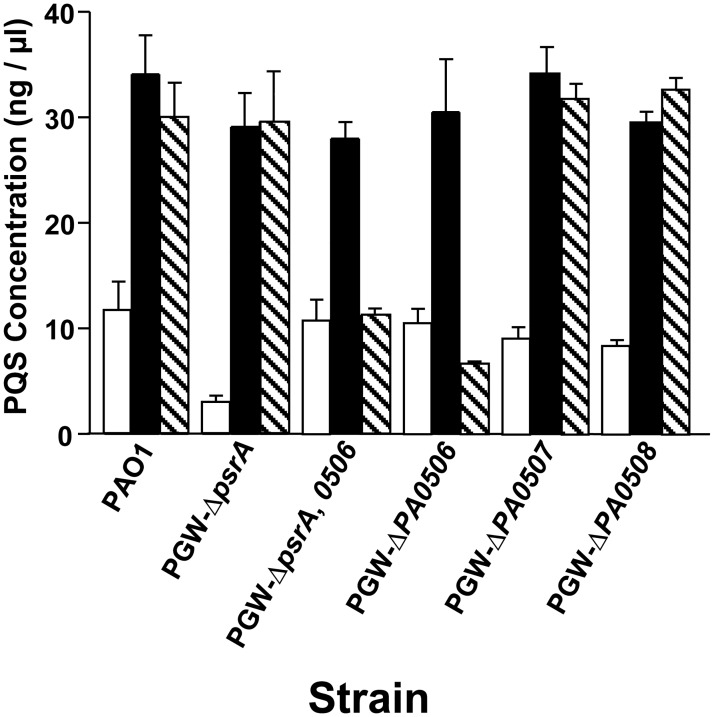
*PA0506* is required for C18 fatty acid to serve as a PQS precursor. Cultures of the indicated strains were grown for 24 h in LB medium alone (open bars) or supplemented with either octanoic acid (black bars) or oleic acid (hatched bars). PQS was extracted and quantified as described in Materials and Methods. Data are presented as the average ± SD of three independent experiments.

PsrA has been shown to bind and repress the *fadBA5* operon, which is important for the breakdown of fatty acids with chain lengths of 12 or greater [21]. This repression by PsrA was also shown to be relieved in the presence of fatty acids, with medium and long chain fatty acids being more efficient at relieving repression than short chain fatty acids [21]. In addition, PsrA has also been shown to bind and repress *PA0506* [19]. This led us to hypothesize that long chain fatty acids would relieve the effect that PsrA had on PQS production. To test this, we grew various *P*. *aeruginosa* strains in the presence of octanoic acid or oleic acid. We found that both fatty acids caused an increase in PQS production in the wild type strain PAO1 and also fully restored PQS production in the *psrA* mutant (Fig 4). Most interestingly, PQS production in the *PA0506* mutant did not increase in response to oleic acid, while the *PA0507* and *PA0508* mutants produced PQS levels that were similar to those seen from the wild type strain grown with oleic acid (Fig 4). These data imply that of the three adjacently encoded FadE homologs, PA0506, PA0507, and PA0508, only PA0506 is important for breaking down long chain fatty acids that will be used for PQS synthesis.

### Overexpression of *PA0506* negatively affects PQS production

**Fig 5:**
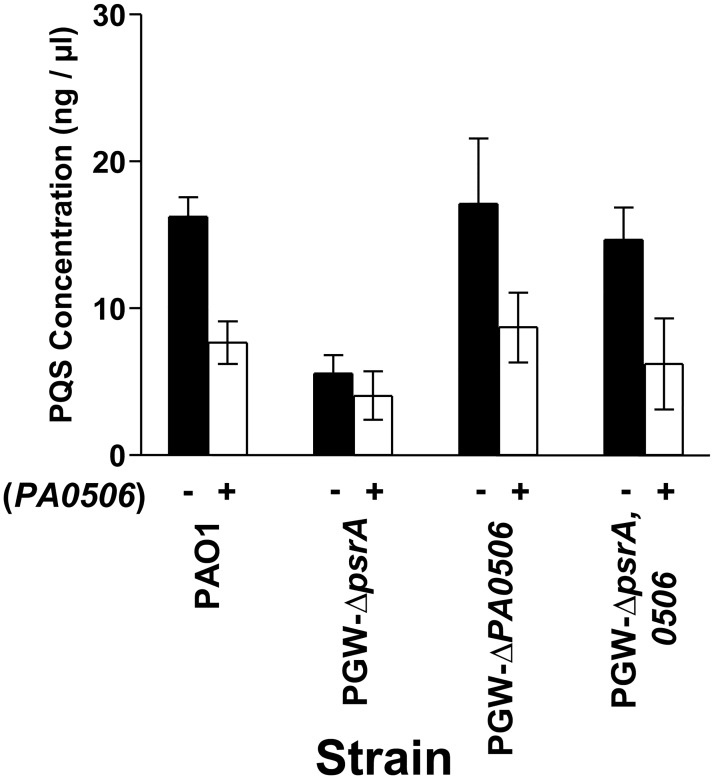
Increased expression of *PA0506* alters PQS production. The indicated strains were grown for 24 h in LB medium harboring pHERD20T (control plasmid) or pGW-21 (*PA0506* expression plasmid) indicated by a minus or plus symbol, respectively. Cultures were supplemented with 0.5% L-arabinose to induce *PA0506* expression, and PQS was then extracted and quantified. Data are presented as the average ± SD of three independent experiments.

The data presented so far caused us to hypothesize that negative regulation of *PA0506* by *psrA* allows normal levels of PQS to be produced and that the decrease in PQS production seen in the *psrA* mutant is due to the increased expression of *PA0506*, which should pull more long chain fatty acids into the β-oxidation cycle, and away from PQS synthesis. To test this idea, we overexpressed *PA0506* in strains PAO1, PGW-*ΔpsrA*, PGW-*ΔPA0506*, and PGW-*ΔpsrA*, *ΔPA0506*. As expected, PQS production was decreased approximately 50 percent when *PA0506* was overexpressed in the wild type strain and *PA0506* mutants (Fig 5). We must note here that the *psrA* mutant containing the *PA0506* expression plasmid had an increase of *PA0506* expression of approximately 8 fold (see S2 Fig), which is less than that seen without a plasmid present (Fig 3A). The reason for this is not clear and we can only speculate that the plasmid and/or the antibiotic selection either increased the expression of our qRT-PCR reference gene or decreased (compared to the strain without a plasmid) expression of *PA0506*. Nevertheless, the data of Fig 5 confirm that the expression of *PA0506* will cause PQS production to decrease. We also determined that when overexpressed from a plasmid, *PA0507* and *PA0508* had a small negative effect on PQS production (S3 Fig). This suggests that these two FadE homologs are capable of changing the supply of octanoyl-CoA, and more importantly, that *PA0506* is the FadE homolog that acts on the precursor needed for PQS to be synthesized.

## Discussion

PsrA is a member of the TetR family of transcriptional regulators that has been shown to regulate numerous genes, including *rpoS*, *PA0506*, and *fadBA5* [19, 41]. It was also linked to PQS production [18] and the data reported here extends these prior studies by showing how PsrA indirectly controls PQS production. PQS production was decreased approximately 85% in a *psrA* mutant (Fig 1), and since *lasR*, *pqsR*, *pqsH*, and *pqsA* were previously identified as genes that controlled the *pqsABCDE* operon [12, 42, 43], we looked at expression of these genes in a *psrA* mutant. Neither *lasR*, *pqsR*, nor *pqsH*, was affected at a level that would produce the observed decrease in PQS production (Fig 2), and these genes, along with *rpoS* were ruled out as potential targets for *psrA*-mediated regulation of PQS. This led us to search for an indirect connection between PsrA and PQS synthesis and the most likely connector seemed to be the fatty acid octanoyl-CoA, which serves as a PQS precursor.

In the fatty acid β-oxidation pathway, FadE catalyzes the conversion of an acyl-CoA to an enoyl-CoA [22]. There are several probable *fadE* genes within the *P*. *aeruginosa* genome, including *PA0506*, *PA0507*, and *PA0508*, and *psrA* has been shown to directly repress *PA0506* [19, 40, 44]. We analyzed the expression of *PA0506*, *PA0507*, and *PA0508* in the *psrA* mutant and found that they were differentially expressed (Fig 3), with *PA0506* expression increasing as seen before [21, 40]. The genetic organization of the genes suggested that they may be in an operon, but their differential expression indicated that they were independently transcribed, which we confirmed (S1 Fig). At this point, we surmised that the overexpression of *PA0506* in the *psrA* mutant could be causing the rapid conversion of acyl-CoA molecules into enoyl-CoA molecules, leaving little precursor for the synthesis of PQS. This idea was strengthened by our data which showed that mutating *PA0506* in the *psrA* mutant led to a restoration of PQS production to a wild type level (Fig 3). Additionally, PQS production was decreased approximately 50% in strain PAO1 expressing *PA0506* from an inducible promoter on a plasmid (Fig 5), which further confirms our hypothesis that *PA0506* repression by PsrA maintains acyl-CoA precursors for PQS synthesis. Mutating *PA0507* or *PA0508* in the *psrA* mutant had no effect on PQS production, and PsrA did not interact with the promoter for either of these genes (Fig 3). Taken together, these data suggested that acyl-CoA molecules that serve as PQS precursors were most likely being processed by the β-oxidation pathway that utilized PA0506 as the enzyme which converts them into enoyl-CoA molecules. Although previous data showed that PsrA also represses the *fadBA5* operon [21], these enzymes act at a later stage in the β-oxidation cycle, after *PA0506* exerts its activity. We found that expressing *fadBA5* from a plasmid had no effect on PQS production (S4 Fig). The reason for this is not obvious but we can speculate that *fadBA5* expression had no effect because an enzyme earlier in the β-oxidation pathway catalyzes the rate-limiting step.

Since a C8 fatty acid serves as a direct PQS precursor, and PsrA is known to regulate fatty acid degradation [16, 19, 21], we investigated whether the addition of medium or long chain fatty acids would restore PQS production in our *psrA* mutant. The addition of octanoic acid to any strain tested caused a major increase in PQS production, even in the *psrA* mutant, indicating that this precursor is greatly limited in this strain (Fig 4). At this point, all of our observations seemed to be pointing to the overexpression of *PA0506* and subsequent loss of acyl-CoA molecules to serve as PQS precursors, as the reason for a decrease in PQS production in the *psrA* mutant. This is interesting because it suggests that PA0506 is important for PQS synthesis from the breakdown of C18 fatty acids but that too much PA0506 is detrimental to the process.

**Fig 6:**
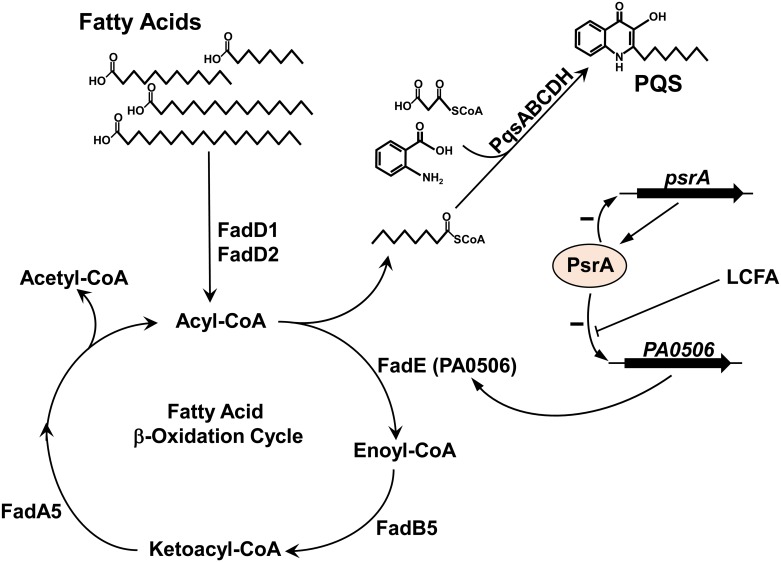
Proposed model of PsrA regulation of PQS production in *P*. *aeruginosa*. In this model, PsrA represses *PA0506* (*fadE*) which allows acyl-CoA to be available for the PQS biosynthesis pathway. Long chain fatty acids (LCFA) relieve this repression. When PsrA is mutated, the expression of *PA0506* is greatly elevated and excess PA0506 will rapidly convert acyl-CoA to enoyl-CoA. This apparently causes a shortage of the octanoyl-CoA needed for PQS synthesis and therefore PQS production greatly decreases.

Taken together, our data suggest that long chain fatty acids that serve as PQS precursors undergo β-oxidation via a pathway that utilizes PA0506, the gene for which is strongly repressed by PsrA. To illustrate this, we present a model (Fig 6) of how the regulation of fatty acid β-oxidation controls PQS production in *P*. *aeruginosa*. It should also be noted here that *P*. *aeruginosa* synthesizes many different quinolones that do not act as intercellular signals but have other physiological functions, and that their synthesis would also be affected by the PsrA-controlled availability of various acyl-CoA molecules. Furthermore, it could be speculated that PsrA-dependent control of the β-oxidation cycle could additionally affect malonyl-CoA availability (another PQS precursor) via altered acetyl-CoA production and this further demonstrates the importance of PsrA regulatory activity during PQS synthesis.

An interesting aspect of this study is that it links PQS production, and thus virulence factor control via cell to cell signaling, to fatty acid degradation via the β-oxidation pathways. These pathways were shown to be induced in *P*. *aeruginosa* growing within the lungs of cystic fibrosis patients where the long chain fatty acids of phosphatidylcholine can serve as a carbon source [5]. *P*. *aeruginosa* β-oxidation pathway mutants were also less virulent in a mouse lung infection model, indicating the importance of fatty acid degradation for virulence in vivo [45]. We speculate that this decrease in virulence is at least partly caused by the dysregulation of quinolone signaling that would occur when the cells can no longer convert long chain fatty acids into the appropriate precursor needed for PQS production.

## Supporting information

S1 Fig Reverse transcription polymerase chain reaction (RT-PCR) analysis of *PA0506*, *PA0507*, and *PA0508* operon structure. Primer locations are indicated by the letters on the gene map and can be matched to the reactions electrophoresed on the agarose gels. For each primer set, lane 1 contains an RT-PCR reaction in which no reverse transcriptase was added (negative control); lane 2 contains a reaction in which chromosomal DNA was added (positive control); and lane 3 contains the experimental reaction.
(TIF)

S2 Fig Control experiment to show expression of *PA0506* from an inducible promoter. qRT-PCR was performed using RNA from strains PAO1 and PGW-Δ*psrA* containing either a control plasmid or one with an inducible promoter that controls *PA0506* (pHERD20T and pGW-21, respectively). All cultures were supplemented with 0.5% L-arabinose except for a control culture of strain PAO1 (pHERD20T), which served as a reference for which the fold change value was set at 1. Data from three independent repeats are presented as average fold change ± SD of expression of *PA0506* as compared to the reference culture.
(TIF)

S3 Fig Effect of expressing *PA0506*, *PA0507*, or *PA0508* on PQS production. Strain PAO1 harboring pHERD20T (control plasmid), pGW-21, pGW-18 or pGW-20 (*PA0506*, *PA0507*, or *PA0508* expression plasmids, respectively) were grown for 24 h in LB medium supplemented with 0.5% L-arabinose. PQS was then extracted and quantified as described in Materials and Methods. Data are presented as the average ± SD of three independent experiments.
(TIF)

S4 Fig Increased expression of *fadBA5* does not affect PQS production. The indicated strains harboring pGW-22 (*fadBA5* expression plasmid) were grown for 24 h in LB medium with or without 0.5% L-arabinose as indicated by a plus or minus symbol, respectively. PQS was then extracted and quantified as described in Materials and Methods. Data are presented as the average ± SD of three independent experiments.
(TIF)

S1 Table Primers used in these studies. (PDF)
