# Supplementary figures and images for: PsrA controls the synthesis of the Pseudomonas aeruginosa quinolone signal via repression of the FadE homolog, PA0506
Source: PLoS One. 2017 Dec 8;12(12):e0189331. doi: 10.1371/journal.pone.0189331 (PMC5722320; doi:10.1371/journal.pone.0189331)

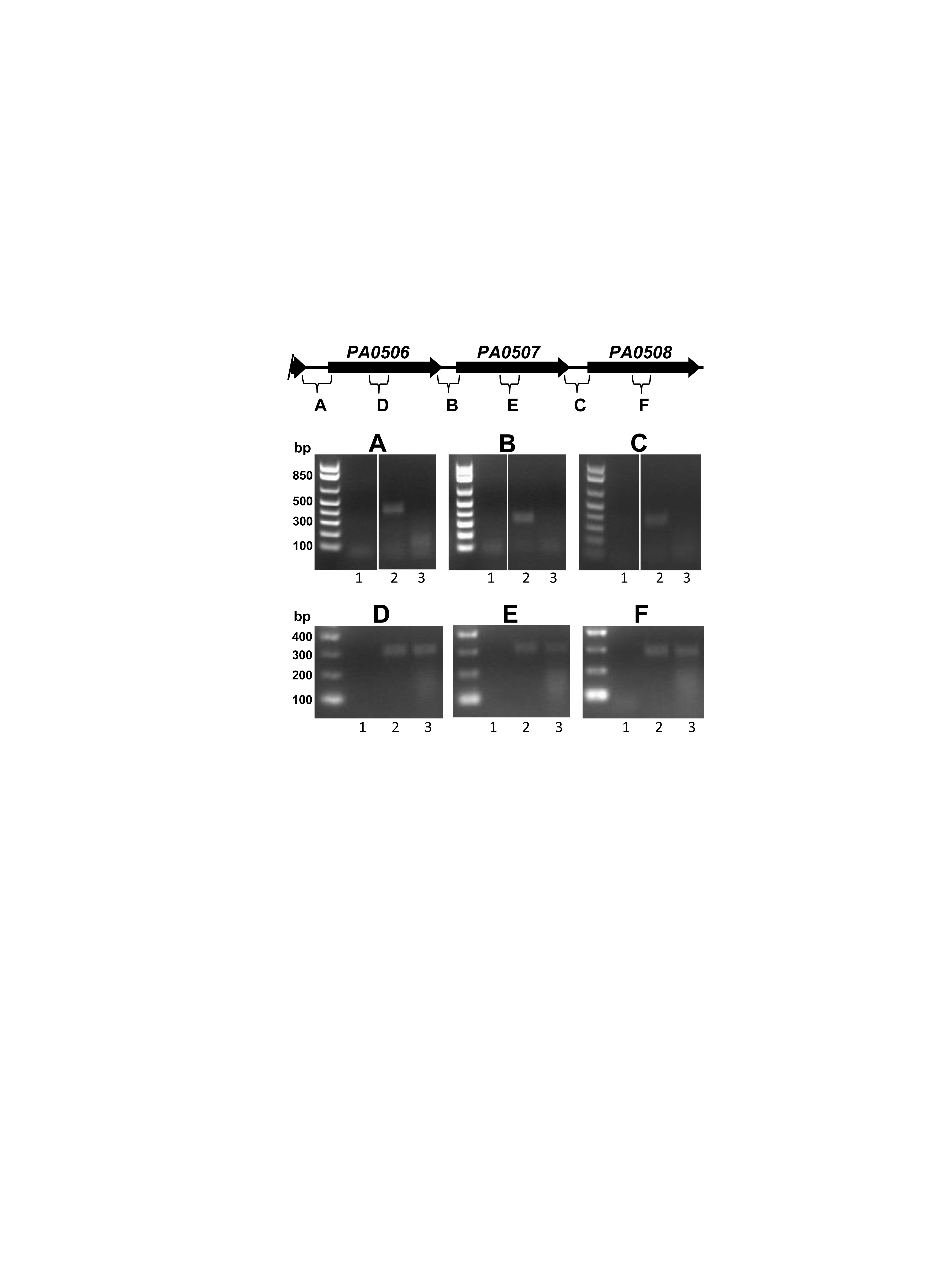

Supplement: S1 Fig — Primer locations are indicated by the letters on the gene map and can be matched to the reactions electrophoresed on the agarose gels. For each primer set, lane 1 contains an RT-PCR reaction in which no reverse transcriptase was added (negative control); lane 2 contains a reaction in which chromosomal DNA was added (positive control); and lane 3 contains the experimental reaction. (TIF) [file pone.0189331.s001.tif]

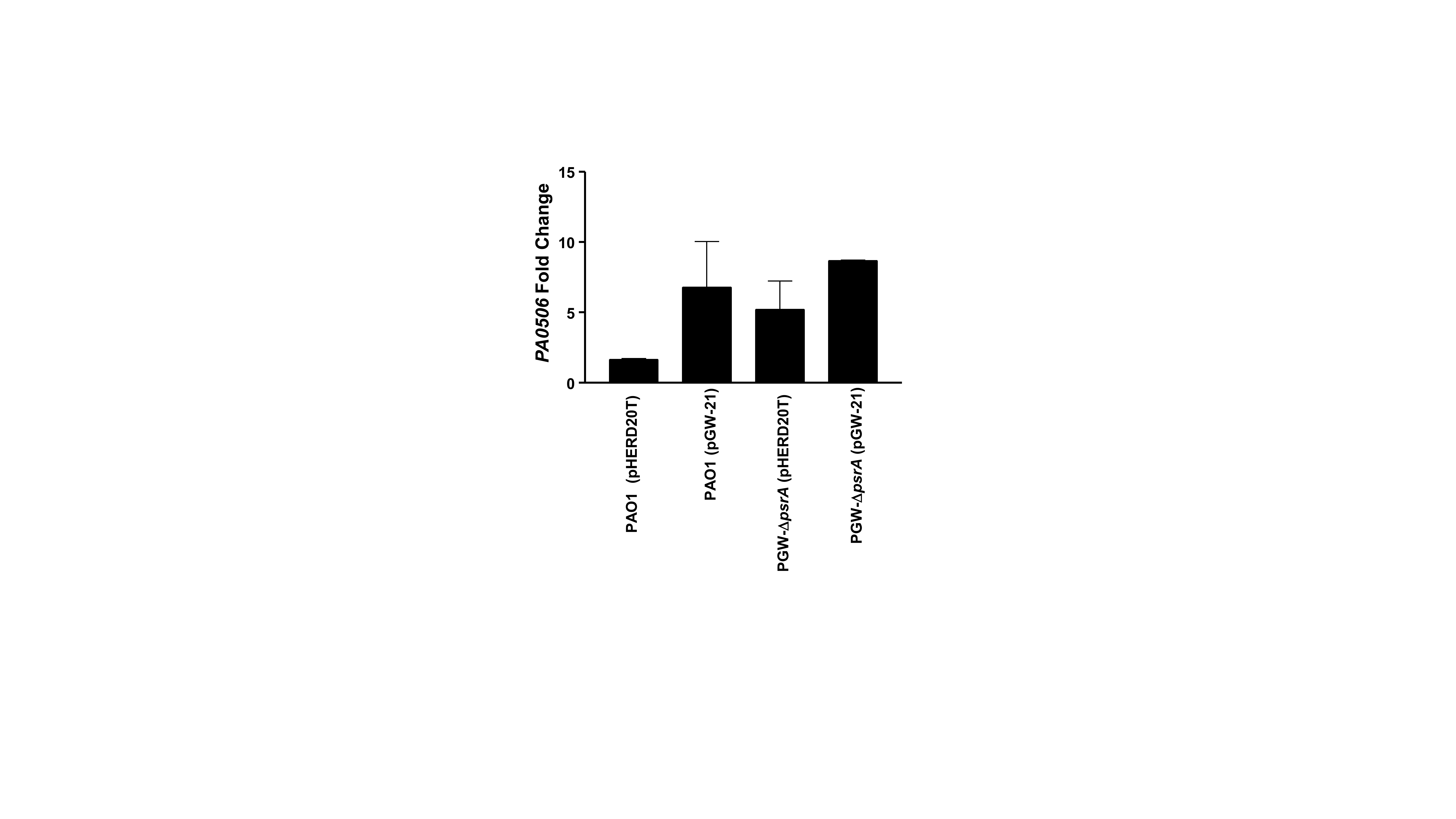

Supplement: S2 Fig — qRT-PCR was performed using RNA from strains PAO1 and PGW-ΔpsrA containing either a control plasmid or one with an inducible promoter that controls PA0506 (pHERD20T and pGW-21, respectively). All cultures were supplemented with 0.5% L-arabinose except for a control culture of strain PAO1 (pHERD20T), which served as a reference for which the fold change value was set at 1. Data from three independent repeats are presented as average fold change ± SD of expression of PA0506 as compared to the reference culture. (TIF) [file pone.0189331.s002.tif]

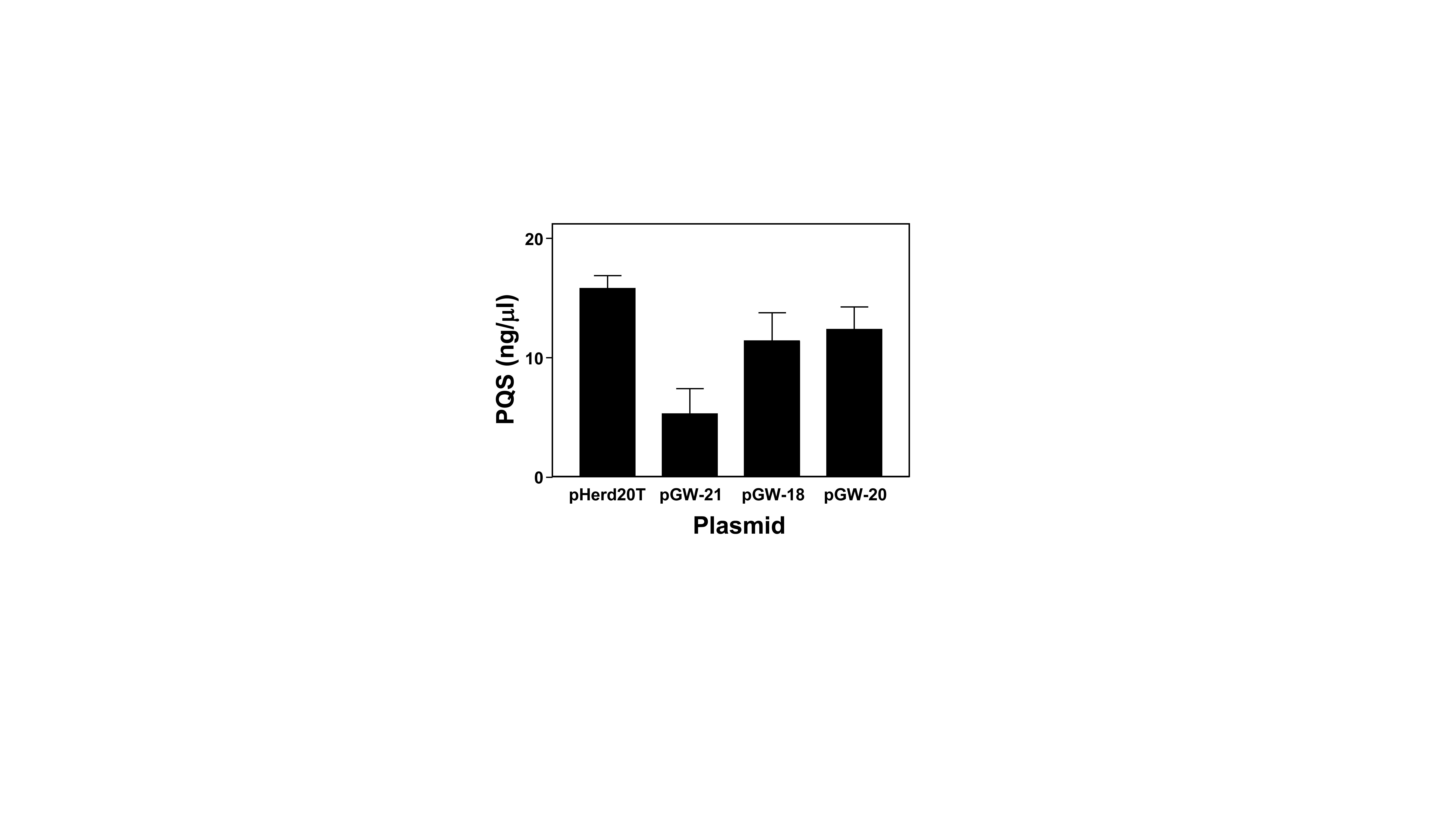

Supplement: S3 Fig — Strain PAO1 harboring pHERD20T (control plasmid), pGW-21, pGW-18 or pGW-20 (PA0506, PA0507, or PA0508 expression plasmids, respectively) were grown for 24 h in LB medium supplemented with 0.5% L-arabinose. PQS was then extracted and quantified as described in Materials and Methods. Data are presented as the average ± SD of three independent experiments. (TIF) [file pone.0189331.s003.tif]

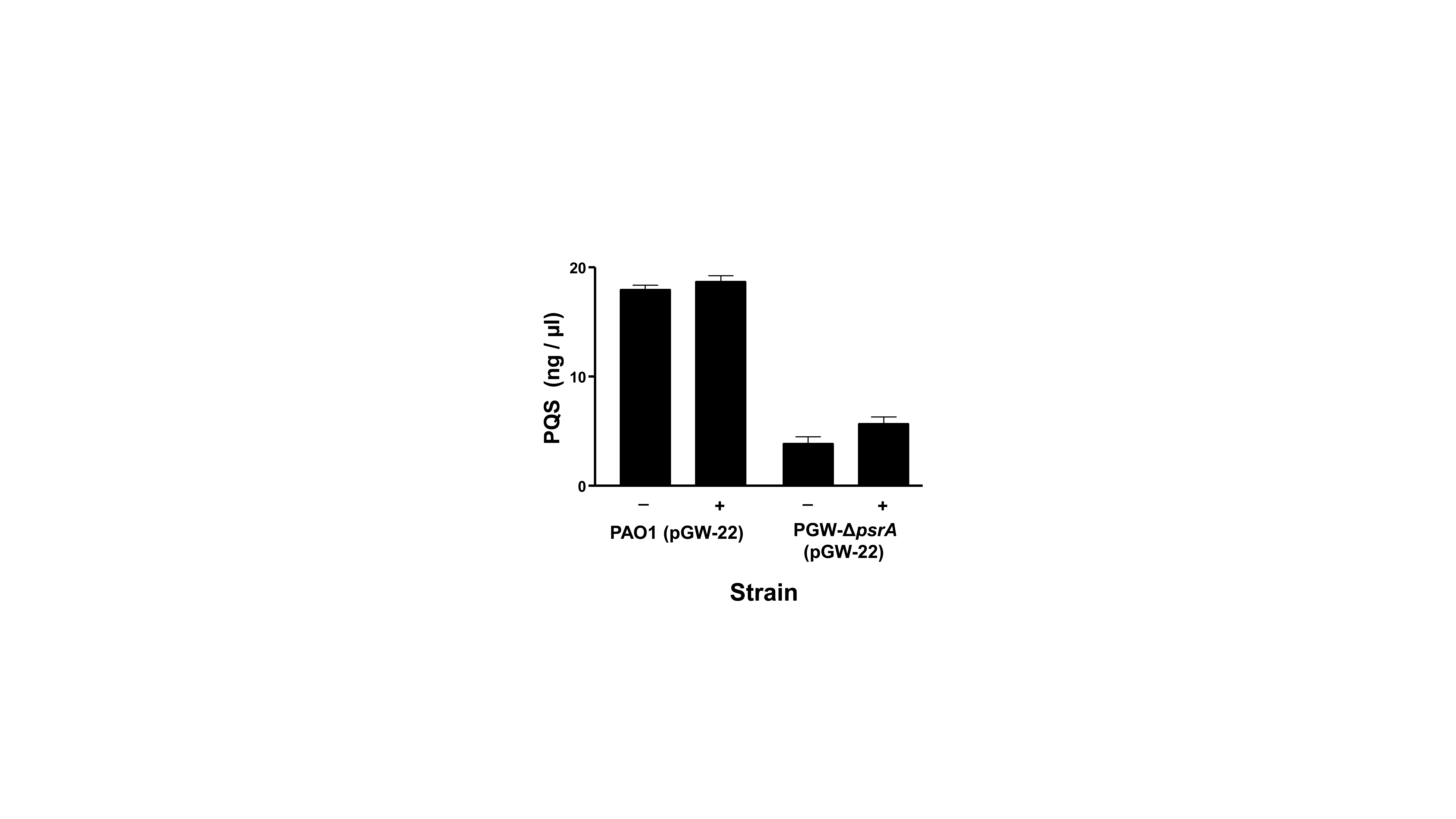

Supplement: S4 Fig — The indicated strains harboring pGW-22 (fadBA5 expression plasmid) were grown for 24 h in LB medium with or without 0.5% L-arabinose as indicated by a plus or minus symbol, respectively. PQS was then extracted and quantified as described in Materials and Methods. Data are presented as the average ± SD of three independent experiments. (TIF) [file pone.0189331.s004.tif]
