# Supplementary material for: PsrA controls the synthesis of the Pseudomonas aeruginosa quinolone signal via repression of the FadE homolog, PA0506
Source: PLoS One. 2017 Dec 8;12(12):e0189331. doi: 10.1371/journal.pone.0189331 (PMC5722320; doi:10.1371/journal.pone.0189331)
Supplement: S1 Table — (PDF) [file pone.0189331.s005.pdf]

Supplemental Table 1. Primers utilized in these studies.

Primer	Sequence (*5'→3'*)[a]
***psrA* Mutation**	
psrA Up1	AAAACTGCAGCTGAATATCACCCCGTCGAACTTCAGC
psrA Up2	CTCGATGCGGCGCAACTGCTGTTCAACAAGACGCCCGCCAAGGCCTGA
psrA Down1	TCAGGCCTTGGCGGGCGTCTTGTTGAACAGCTGTTCCGCCGCATCGAG
psrA Down2	AAAACTGCAGCTGTCGATGACATCGTCGAGCAAGGGT
**PA0506 Mutation**	
0506 Up1	AAAAAAAGCTTGCTGTATCAACCATGCGCTG
0506 Up2	CTCCATCAGGTTGTTGGCCAGCAGCTCGTCGCGAAC
0506 Down1	GTTCGCGACGAGCTGCTGGCCAACAACCTGATGGAG
0506 Down2	AAAAAAAGCTTGGCCAGCACCAGGTGGACGAT
**PA0507 Mutation**	
0507 Up1	AAAAAAAGCTTATCATCGTGCATCCTGACG
0507 Up2	CTTGGCTTCGTAGAACGCGTCCGGGGTCGCGTCGCT
0507 Down1	AGCGACGCGACCCCGGACGCGTTCTACGAAGCCAAG
0507 Down2	AAAAAAAGCTTGGTGATGAAGATCTTGGT
**PA0508 Mutation**	
0508 Up1	AAAAAAAGCTTTACTTCACCGCCCAGTTGCTCG
0508 Up2	GGTGCGCGCCATCAATGCCTCGGCGTCGACCACTTC
0508 Down1	GAAGTGGTCGACGCCGAGGCATTGATGGCGCGCACC
0508 Down2	AAAAAAAGCTTGCCTCAACACGCGCAACCTGG
**Gene Expression**	
PsrA Up	AAAAGAGCTCTCAGGCCTTGGCGGG
PsrA Down	AAAACCATGGCCCAGTCGGAAACC
PA0506 Up	AAAAAGAGCTCATGCCTGATTACAAGG
PA0506 Down	AAAAAAAGCTTTCAGTAACCGAGGGCGAA
PA0507 EXPR F	AAAAAGAGCTCATGCCGGAATACAAGGCT
PA0507 EXPR R	AAAAACTGCAGCTAGAGCGAAAACTGCTCCGCCGA
PA0508 EXPR F	AAAAAGAGCTCATGGCTGATTACAAAGCTCCG
PA0508 EXPR R	AAAAACTGCAGTCAGAACTGCTCGGCGTCCAG
FadAB5 EXPR F	AAAAAAAGCTTTTAGATGCGCTCGAAGACGG
FadAB5 EXPR R	AAAAAGAGCTCATGATTTACCAAGGTAAAG
**RT-PCR**	
PA0505 RT-F	GTCATCGAGGTAGCCGACGAGA
PA0506 RT-R	TGCGCTTCGTAGCCCAGCAGCTCG
PA0506 RT-F	ATGCTGTCCGGCGCCAACAACCTGAT
PA0507 RT-R	GTCGAATACCTCATCGATCAGGA
PA0507 RT-F	GGCGGGCCTGATGAGCCTGTCGGCG
PA0508 RT-R	AGAGCCGGGAGACCTCGAACA
PA0505 RT-C-F	GGCACCAAGATCTTCATCTCCG

PA0506 RT-C-R	GAAGGTGAACATGCAGTTCAGGC
PA0506 RT-C-F	GAAGATATTCATCTCCGCCGGCGAG
PA0507 RT-C-R	GGCGTGGTTCATCATGGTGAACATG
PA0507 RT-C-F	GCGATGTTCACCATGATGAACTACGAGCG
PA0508 RT-C-R	GTCTTCGCTGTACTTGGCGGTGTCCAG
**EMSA**	
psrA promoter F	AAAAAAAAGCTTCAGCTGTTCCGCCGCATCGA
psrA promoter R	AAAAACTCGAGGTCGTCGCTCTGTGTTTTTA
kynA promoter F	GAGTGAGGGCAAGGACACAT
kynA promoter R	CGCGAGTGATCCGAAATTCG
PA0506 EMSA F	GACCCTTCCTTTTCCGACTGTCA
PA0506 EMSA R	GAAACGGATATCACGCAA
PA0507 EMSA F	AGGTGAGAAAGAGCCCGCCCTT
PA0507 EMSA R	CTCATCGATCAGGAAGCGCATGTC
PA0508 EMSA F	GCTCAGCGGCTGCCGGGGCAG
PA0508 EMSA R	GACCTCGAACACCTCATTGAG
***psrA* sequencing**	
psrA seq F	ACGGGCCTCAGACAGCGCAC
psrA seq R	ACGGGCCTCAGACAGCGCAC
**Quantitative Real Time-PCR**	
PqsA rt 1	GCCCTTTGCTCGACGATTTCTCG
PqsA rt 2	AACCCGAGGTGTATTGCAGGAAACA
LasR rt 1	CCTTCATCGTCGGCAACTA
LasR rt 2	GGACGGTTCCCAGAAAATC
PA0506 rt 1	CTTGCGTGATATCCGTTTC
PA0506 rt 2	CACCTGTTCGCAGAACTT
PA0507 rt 1	GATGAAGGGTCGAAATTCTG
PA0507 rt 2	GTGCATAGGCTTCCTTGAAA
PA0508 rt 1	GTCGAGGAACTGGTCAACTC
PA0508 rt 2	CAGGTACTTGTCCTTCAGTTC
ClpX rt 1	CCTGTGCAATGACATCATCC
ClpX rt 2	AGGATGGTGCGGATCTCTTT
PqsH rt1	ATGCCGTTGGCCGGCATCAA
PqsH rt 2	ACGATGGAGACCCCGACCCG
PqsR rt 1	ATTGCGCGGACCCTTGTTGA
PqsR rt 2	GGACGGCTACAAGGTCGAAC

[a]Underlined sequence denotes restriction site utilized for cloning.
